# Supplementary material for: Multiscale dynamical network mechanisms underlying aging of an online organism from birth to death
Source: Sci Rep. 2018 Feb 23;8:3552. doi: 10.1038/s41598-018-22027-z (PMC5824793; doi:10.1038/s41598-018-22027-z)
Supplement: Supplementary file 1 — Supplementary information [file 41598_2018_22027_MOESM1_ESM.pdf]

Supplementary Information (SI) for

Multiscale dynamical network mechanisms underlying aging of an online organism from birth to death

M. Zheng[1,2], Z. Cao[1,2], Y. Vorobyeva[3], P. Manrique[1,2], C. Song[1,2] and N.F. Johnson[1,2]

[1]Department of Physics, University of Miami, Coral Gables, FL 33146, USA

[2]Complexity Initiative, University of Miami, Coral Gables, FL 33146, USA

[3]Department of International Studies, University of Miami, Coral Gables, FL 33146, USA

Contents:

Data analysis (p. 2)

Additional details of relevant neuroscience literature (p. 6)

Additional details of results from data analysis (p. 8)

Other Supplementary Information for this manuscript includes the following:

Movie of evolving organism bipartite network, in vivo and in continuous time throughout lifespan. *(Since the movie file for the entire organism is too big to upload, the current movie upload is a random sample of 1000 nodes (1000 users) from just before maturity until old-age -- just as an illustration).*

The datafile DataForFigures.xlsx which contains the data for the figures.

Data analysis

Data collection

Just as occurs commonly on Facebook where individuals with a common interest in a particular activity form an online Facebook group, the data shows that operational pro-ISIS and protest narratives develop through self-organized online groups, each of which is an ad hoc group of followers of an online page created through ВКонтакте (VKontakte) at www.vk.com. The generic web-based interface allows such online groups to form in a language-agnostic way, and with freely chosen names that help attract followers without publicizing their members' identities. Facebook almost immediately shuts down online groups if their content includes material promoting pro-ISIS extremism. Online groups on VKontakte experience longer lifetimes, perhaps because predators (online moderators) either have more trouble finding them or have less available resources for searching. The precise reasons, while interesting, do not affect the analysis in our paper. Because the focus in this paper is on the entire organism (i.e. the self-organized body of online pro-ISIS support) rather than the behavior of any individuals, no names are being released. Pro-ISIS online groups (i.e. *functional units*) inhabit an online environment in which predatory entities (website moderators) seek to shut down pro-ISIS activity and narratives. In contrast to the largely mundane chatter that may casually mention ISIS on Twitter and in online groups focused on sport for example, pro-ISIS online groups frequently discuss operational details such as routes for financing, technological know-how and avoiding drone strikes. We chose VKontakte for our pro-ISIS analysis as (i) pro-ISIS online groups are shut down essentially immediately on Facebook, but not on VKontakte; (ii) it is the largest European online social networking service, with more than 350 million users; (iii) it allows multiple languages and is used worldwide; (iv) being based in Russia, it has a high concentration of users of Chechen origin focused in the Caucasus region near ISIS' main area of influence in the Levant; (v) ISIS used it to spread propaganda among the Russian-speaking population. Our methodology for identifying these pro-ISIS online groups was as follows. We manually identified relevant narratives using hashtags in multiple languages, e.g. *#isn #khilafah #fisyria* #игиш (i.e. ISIS) #дауля (i.e. *dawla*, meaning 'state') #халифат (i.e. 'Caliphate'), and traced these to underlying online groups. The specific criterion for inclusion in the list was that the online group explicitly expressed its support for ISIS, publishing ISIS-related news or propaganda and/or calling for jihad in the name of ISIS. This list was fed into software Application Programming Interfaces (APIs) that expanded it by means of automated search snowballing. The expanded online group list was then cross-checked to eliminate false identifications. New embedded links were manually searched to identify more online groups and hashtags. We then iterated this process until closure of the online group list (i.e. the search led back to online groups that were already in the list). Although labor intensive, we were able to find closure on a daily basis in real time. The manual list of online groups for each day was assembled by the SMEs (Subject Matter Experts) to include only those appearing to express a strong allegiance to ISIS. The SMEs updated this manual list every day by logging into VK.com on a daily basis at the same time of the day, and manually searching for newly created online groups by a)

analyzing posts and reposts within the known online groups; b) following selected profiles that actively publish ISIS news as well as analyzing the online groups they followed, if any. Whenever a new online group name was found, it was analyzed to establish the relevance of its narrative content and then -- if appearing to be relevant as judged by the subject-matter experts to the best of their abilities – that online group was included in the database. Once the online groups supporting ISIS were identified in this way, an additional search using their followers and online groups to whom they linked was performed on that same day. Thus, the manual content analysis helped identify new online groups created -- and also those that had been shutdown. An example of this manual list of online groups is available on request from the authors. Though this list is subject to human error as with any human task, the initial manual approach in our methodology using subject matter experts was crucial since online groups are a commonly occurring phenomenon on sites like VKontakte, but the vast majority of these online groups have nothing to do with pro-ISIS activity and so the pro-ISIS ones can be lost in the sea of otherwise innocuous online groups. Unless the pro-ISIS online groups are noticed, and the finding agent (predator) shuts them down, they will continue to live within this sea of other online groups. As mentioned earlier, Facebook almost immediately shuts down such pro-ISIS online groups, but on VKontakte they experience longer lifetimes, perhaps because predators either have more trouble finding them or have less available resources for searching. There can be additional costs to shutting down online groups, apart from the time and effort spent, since it may make for bad press with its followers claiming freedoms of expression are being compromised by that predatory entity. We note that online groups are technically distinct from the conventional network science definition of hubs where a particular node (e.g. person) has a large number of links. For an online group, by contrast, a number of followers actually aggregated together without any one of them necessarily having many more links than the others. Our analysis therefore goes beyond existing approaches to mining online data, by uncovering a new collective human behavior in the online world comprising self-organized online groups under pressure. Faloustos et al. (D. Koutra, D. Jin, Y. Ning, C. Faloutsos, Perseus: An interactive large-scale graph mining and visualization tool (DEMO), VLDB 2015, Kohala Coast, Hawaii, 31 August to 4 September 2015) focus on pattern discovery (e.g. fractals) in time-evolving multi-aspect data while Leskovec et al. (Q. Zhao, M. Erdogdu, H. He, A. Rajaraman, J. Leskovec, SEISMIC: A self-exciting point process model for predicting tweet popularity, ACM SIGKDD International Conference on Knowledge Discovery and Data Mining (KDD), Sydney, 10 to 13 August 2015) look at information flow and antisocial behavior in online communities and Tweets. Vespignani et al. have analyzed the evolution and diffusion in networks and sub-networks, including Twitter (see http://www.mobs-lab.org/alessandro-vespignani.html). Shakarian and co-workers (A. Stanton, A. Thart, A. Jain, P. Vyas, A. Chatterjee, P. Shakarian, arXiv:1508.01192v1 (2015)) are working toward measurement and analytic technologies for detecting information cascades and understanding the ideological orientations of participating communities, while a separate work of theirs analyzes ISIS activity on the ground using an incident dataset. Our analysis is distinct in its analysis and findings concerning collective online group dynamics among ISIS supporters, and in its methodology which combines (i) automated

data-mining with (ii) subject-matter expert analysis, together with (iii) generative model building drawn from the physical and mathematical science community. It is this triple combination of tools that enables us to discover, unravel and quantify the self-organized entity of online pro-ISIS support lying beyond any background buzz. A detailed primer video showing how online groups can be set up in general (i.e. for any purpose) on the generic interface used by VKontakte etc. is provided at: https://youtu.be/pcxu8yHdBUM. An advantage of this setup for supporters of an entity such as ISIS, is that the online group name need reveal nothing about individuals' identities. The online user platform of VKontakte is very similar to that of Facebook and other social media sites, making our methodology transferrable across languages and continents. An example of a screenshot of an online group on VKontakte (ВКонтакте) is available on request from the authors. We developed web analysis software to collect additional information daily surrounding the pro-ISIS online groups from the manual list, including who was in the online group (i.e. follower), the posts in the online group, plus Boolean variables to denote whether or not the online group had been shutdown or not. The software collected this information automatically on a daily basis and stored it in a flat-file database on private servers. Given that pro-ISIS sites may be shut-down on VKontakte, our collection scripts not only allowed us to pool information about ISIS online groups from vk.com but also find previously disabled groups that may have re-emerged under a different name. All the data for the pro-ISIS activity were from VKontakte. The building of the list of online groups was stopped when the iterative search process described in the main paper, led back to online groups that were already in the list and hence we reached closure. If not provably complete, we believe that our list was at least highly representative of the full ecology of relevant online groups – subject of course to the limitation that it is impossible to establish 100% the true intent of an online group and hence judgments needed to be made by the human subject-matter experts regarding exclusion or inclusion. At no stage did we go beyond accessing open-source information -- nor did we employ any technology or software that is beyond the level of that currently used routinely in the academic field of web and social media analysis. The following examples are illustrative of the content of the pro-ISIS online groups that we identified. Specific web-links are available from the authors for each:

1) Evidence of fundraising: Multiple incidents of collecting funds for potential fighters who want to travel to Syria but cannot afford it. Also transfer of funds for fighters who are already in Syria.

2) Evidence of real-time operational information stream: Some online groups resemble an alternative news outlet where they stream information directly from their territory. Operational updates from battlefield, e.g. the specifics of the Kobane radio tower in real-time. One example image that we uncovered, which is available from the authors, says "ISIS took control over the Kobane's radio tower"; "Mujahedeen advanced 500 meters into Kobane".

3) Evidence of mobilizing support: Images available from the authors, include text such as: "Brothers! Yesterday, in a German town of Celle, a 100-people mob of Yazidi Kurds beat up 5 Chechens in retaliation for Chechens fighting within ISIS in Iraq and Syria where they kill Kurds. Since Celle has the largest Yezidi Kurds community (about 5000 people), our local brothers' lives are under threat. This is a call to all brothers from

nearby locations to send groups of 30-40 people to protect our brothers in distress!". By comparison, a real news report of the events appears at the following link: http://www.jamestown.org/programs/edm/single/?tx_ttnews%5Btt_news%5D=42950&cHash=a3d9f3712a5af1132bbada1cc2466f34#.Vk7b3XarTIU

This news report shows that the online group had not invented the story, i.e. the online group is indeed operationally relevant. The article provides evidence of a real social mobilization through social media/online activity. Another screenshot says: "Urgent! Those who are travelling to Celle for the fight, do NOT go! The Kurds have apologized and there will be a peaceful settlement. German border authorities are not allowing Chechens enter from Austria, France and other countries. All German cities are full of police patrols." It was published a day after the previous one.

4) Teaching survival skills: Some pro-ISIS online groups include advice on cellphone and Internet use during an operation in order to avoid being detected by security services; and also ways to prevent or repel a drone attack during an operation.

5) Recruitment: Evidence that the online groups serve as a platform to spread recruitment messages, is illustrated by an example available from the authors, that states: "IS fighters in Dagestan call other Caucasus mujahedeen enter their ranks". Indeed many Caucasus guerrilla groups joined ISIS later.

Coalescence-fragmentation model leading to expression in manuscript for the time for elimination T_e

This analysis builds on the model of the online group dynamics presented in the 2016 *Science* paper by Johnson et al., referred to in the main paper (Ref. 34). We assign each functional unit (i.e. online group) a specific label $i,j,k,\ldots$. Each has a certain number of *follows* at a given timestep, denoted by $s_i, s_j, s_k, \ldots$ respectively. We take the sum of the follows over all online groups to be equal to N where $\sum_{i,j,k,\ldots} s_i = N$. The total number of follows N is therefore being dynamically distributed among online groups (or left as unattached) as a result of an ongoing process of aggregation (coalescence) and fragmentation. Consider an arbitrary online group i with size s_i. At any one instant in time, labelled t, this online group may either:

- Get attacked by the opposing entity such as online moderators. The number of follows in that original cluster is then assumed to be reduced since follows are destroyed. We have implemented multiple variants of this scheme, and have checked that the main feature of a peak in T_e still appears at high asymmetry. See for example, the Phys. Rev. Lett. 2009 paper referred to below.
- Coalesce (i.e. combine) an unattached follow to an online group, or (as we also observe occurs in the real data) an online group gets absorbed into another online group j of size s_j, hence forming a single online group of size $s_i + s_j$. We stress that an online group of size 1 is simply another way of saying an isolated follow -- hence when we talk about coalescence of two online groups, this includes the possibility of addition of an isolated follow. Indeed since there are so many isolated follows, the data shows that the addition of an isolated follow is a common process.

For more details, we refer to:

"Statistical Physics and Modern Human Warfare", in *Mathematical Modeling of Collective Behavior in Socio-Economic and Life Sciences*, Eds. Naldi et al., Birkhäuser Boston (2010), DOI: 10.1007/978-0-8176-4946-3_14 p.365; *Alex Dixon, Zhenyuan Zhao, Juan Camilo Bohorquez, Russell Denney and Neil Johnson*

"Common ecology quantifies human insurgency", Nature 462, 911 (2009); *Juan Camilo Bohorquez, Sean Gourley, Alex Dixon, Mike Spagat and Neil Johnson*

"Anomalously slow attrition times for asymmetric populations with internal group dynamics", Physical Review Letters 103, 148701 (2009); *Zhenyuan Zhao, Juan Camilo Bohorquez, Alex Dixon and Neil F. Johnson*

More details of relevant neuroscience literature

In this section, we discuss more details of the modern understanding of networks in neurological science and aging, focusing on the work of Sporns and Witten and their co-workers (see references in the main paper) and using their quotations to present current thinking, in order to help avoid any misunderstanding introduced by our paraphrasing.

As noted by many researchers -- including Sporns, Bullmore, Seung, Lichtman and co-workers (see references in main paper) – the conservation of resources alone is insufficient to explain all aspects of brain network architecture. Not only have long-distance connections been evolutionarily conserved, they have been expanded in cases where this expansion has generated increased network performance. As stated in Sporns' classic reference (O. Sporns. Networks of the Brain (Kindle Locations 2769-2773)): "The topology of structural and functional brain networks changes profoundly during neural development, adulthood, and senescence, due to a multitude of mechanisms for growth and plasticity, operating on different cellular substrates and time scales." This is exactly what we see in our organism's data. As Sporns also states: "Disturbances of these mechanisms are potential candidates dates in a variety of neurodevelopmental disorders, and they highlight the delicate balance that must be maintained for properly organized connectivity to emerge. One of the most puzzling aspects of structural brain networks at the cellular scale is their extraordinary propensity for rewiring and remodeling in the presence or absence of neural activity. Some studies suggest that individual synapses can change shape and even come into and go out of existence on time scales far shorter than those of some forms of memory. Many elements of the brain's "wiring pattern," or structural connectivity graph, at the scale of cells and synapses, appear to be highly dynamic, and the relative instability of individual synapses casts doubt on their reliability as sites of long-term modification and memory. If these observations are further extended and found to generalize across much of the brain, then processes that ensure some degree of stability or "functional homeostasis" at the level of the entire system will become of central importance. Processes of self-organization also appear to underlie the highly variable able yet

robust nature of brain dynamics. It turns out that mechanisms of plasticity may play an important role in maintaining the networks of the brain in a dynamic regime that ensures high sensitivity to inputs, high information capacity, and high complexity." As also emphasized by Sporns et al in 'The Human Connectome: A Structural Description of the Human Brain', in PLoS Computational Biology by Olaf Sporns, Giulio Tononi, Rolf Kotter. 1, e42, 0245 (2005): "To truly understand the functioning of any complex system, one must know not only its elements and their interconnections, but also how they change over time".

In terms of what is known about aging and networks, Witten has explained in detail that "since the creation and maintenance of newly established links between two network elements are costly, and aging is usually accompanied by a decline in the system's resources, aging generally induces a loss of links, leading to a declining network." This is seen in our data by measures such as the number of users and follows decreasing, together with a decrease of modularity (clustering coefficient) for the functionome. There is also a loss of small-world-ness in the functional network meaning that elements cannot find each other so easily in the aged network. As also stated by Wimble and Witten in "Applications to Aging Networks" in Yashin AI, Jazwinski SM (eds): Aging and Health – A Systems Biology Perspective. Interdiscipl Top Gerontol. Basel, Karger, 2015, vol 40, pp 18–34 (DOI: 10.1159/000364925): "While little is currently known about how aging-related networks evolve across the organism's life span, it is reasonable to assume that two possible changes can occur; in-activation of active nodes/activation of inactive nodes and loss of connectivity/increase in connectivity. How or why nodes become inactive or edges disappear is irrelevant here; just that they do. It turns out that the structure of small-world networks, due to their hub connectivity, makes them vulnerable to targeted attacks aimed at specific hubs. Attacks that knock out essential genes are knocking out the life span network because the organism dies when an essential gene is knocked out. Thus, essential genes are critical hub genes. Small-world neural networks have been shown to exhibit short-term memory capability. This suggests that memory decay, such as that seen in Alzheimer's disease (AD) may be related to decay of brain neural network structure in such a way as to remove the small-worldness property of the memory network. Understanding patterns in network decomposition could lead to potential early AD detection and to potential pharmaceutical intervention at earlier points in the disease course…. Connectivity gain and loss also have implications when it comes to discussing the hierarchical modularity of aging-related network architectures. Loss of connectivity through inactivity of a node or through loss of an edge could unlink an entire module of importance. Thus, nodes that connect modules within a larger network are critical to the functioning of the network. Questions around the role of evolutionary processes in the development of network architectures of various organisms may be of importance in understanding how network architectures related to aging processes are constructed…Why are some components of a network redundant while others are not (see also all of the citations on reliability theory)? What is the role of backup subnetworks? What is the importance of robustness and resilience? Why are some networks more robust to attack, less fragile than others or morefrail? How do we balance the need to adapt and evolve with robustness? What, if any, is the association of life span with network architecture?" We believe that the use of a proxy organism

dataset as in the main paper, can help answer these questions and others.

Additional details of results from data analysis

For the growing stage ($0<t<70$) in Fig. 1(a), the number of groups grows fast and is almost proportional to the growth of number of follows, which results in very slow growth of average group size. This indicates a high birth rate of new groups. Users in this stage has no strong preference of which group to join, and are instead active in trying to join diverse groups. This is shown in Fig. 1(a) in that all groups are close to each other during the growth phase. At this stage, the system is forming some sub-networks—which are 'groups of groups' or communities of communities. The number of follows per user (and per group) is high, respectively, and the degree assortativities are negative while the clustering coefficients are low for the user-projected network. The density of the group-projected network is high and the average path length is relatively short. This suggests that most of the groups are still young and any preferential-attachment-like advantage has not yet been built up. In addition to actively initiating new groups, users in this stage are also most active in writing posts, and the productivity, defined as number of new posts (each day) per follow (or alternatively, per user), becomes saturated in this stage very quickly because the number of follows per user in this stage has been in the range of around 2.25-2.5, which corresponds to several of the peaks in Fig. 3. When the system evolves to the mature stage ($70<t<110$), the number of groups as well as number of follows per user reach their peaks, average group size keeps growing. The total number of follows keeps growing, which results in more follows and less groups and therefore high density for the group-projected network, but low density (or a peak on path-length) for the user-projected one. In this stage, large groups with special interests are formed (shown in Fig. 1b as the appearance of remotely connected large groups), in which most members are strongly preferentially attached to the group they joined. This is further supported by a decreased PA (preferential attachment) exponent for the CCDF of group size. At this stage, the communities became stronger than the former stage. As for posts, we noticed that number of new posts and that per user or per follow all decrease; furthermore, the number of follows per user increased, and therefore we infer that the productivity has run into post-saturation stage. With respect to the dying stage ($t>110$), both number of groups and number of follows per user decay with time; however, both average group size and number of users keep growing. Natural aging in this connectome also occurs, with individual users (nodes) occasionally shutting down their own accounts after they had had enough of the online extremist experience, just as a neuron or more general network node might die over time. The same is true of their links, just as an individual synapse in the brain might cease to be operational. As a result of these mechanisms, the organism had started to die off by timestep 250 with activity per user all but disappearing toward the end of 2015. Eventually there were far less functional groups, and the ones that existed showed hardly any activity. What those users went on to do (e.g. inhabit more secretive online worlds such as Telegram or Tor) is tangential to our study. The figure below shows the sharp increase in the fraction of nodes involved in just one functional unit:

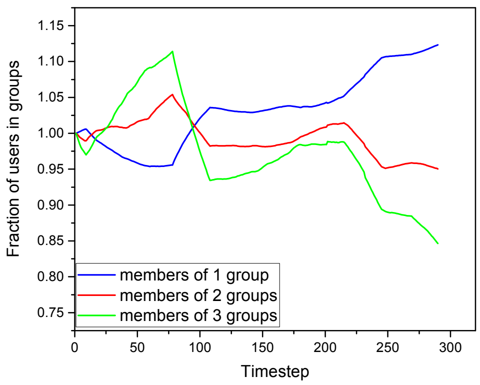

Fig. S2

Increase in the fraction of nodes involved in just one functional unit. Curves are averaged over a short time-window to emphasize the trends.

Impact of one-off intervention (Fig. 4)

$H' = \text{alpha } H$

alpha	t_I	%
0.829	80	10
0.502	80	30
0.306	80	50
0.867	120	10
0.609	120	30

$Tm' = \text{beta } Tm$

beta	t_I	%
1.032	80	10
1.01	80	30
---	80	50
0.968	120	10
0.813	120	30

$T' = \text{gamma}\ T$

gamma	t_I	%
1.014	80	10
1.08	80	30
1.12	80	50
1.001	120	10
1.019	120	30
0.961	120	50
0.991	200	10
0.94	200	30
0.858	200	50
